# ERGA-BGE reference genome of *Leviellus thorelli, *a common orb-weaving spider representing the *Zygiellidae* family

**DOI:** 10.12688/openreseurope.21671.2

**Published:** 2026-02-10

**Authors:** Matjaz Gregorič, Elena Bužan, Astrid Böhne, Rita Monteiro, Rosa Fernández, Nuria Escudero, Marta Gut, Laura Aguilera, Francisco Câmara Ferreira, Fernando Cruz, Jèssica Gómez-Garrido, Tyler S. Alioto, Chiara Bortoluzzi

**Affiliations:** 1 Research Centre of the Slovenian Academy of Sciences and Arts, Novi trg 2, Jovan Hadži Institute of Biology, Ljubljana, 1000, Slovenia; 2 Postgraduate School ZRC SAZU, Novi trg 2, Ljubljana, 1000, Slovenia; 3 Faculty of Mathematics, Natural Sciences and Information Technologies, Glagoljaška 8, University of Primorska, Koper, SI-6000, Slovenia; 4 Faculty of Environmental Protection, Trg mladosti 7, Velenje, 3320, Slovenia; 5 Leibniz Institute for the Analysis of Biodiversity Change, Museum Koenig Bonn, Adenauerallee 127, Bonn, 53113, Germany; 6 Metazoa Phylogenomics Lab, Passeig marítim de la Barceloneta 37-49, Institute for Evolutionary Biology (CSIC-UPF), Barcelona, 08003, Spain; 7 Centro Nacional de Análisis Genómico (CNAG), Baldiri Reixac 4, Barcelona, 08028, Spain; 8 Universitat de Barcelona (UB), Barcelona, 08028, Spain; 9 SIB Swiss Institute of Bioinformatics, Amphipôle, Quartier UNIL-Sorge, Lausanne, 1015, Switzerland

**Keywords:** Leviellus thorelli, genome assembly, European Reference Genome Atlas, Biodiversity Genomics Europe, Earth Biogenome Project, Zygiellidae, free sector orb weaver

## Abstract

The *Leviellus thorelli* reference genome provides the first high-quality genomic resource for *Zygiellidae*, a family of orb-weaving spiders with a dynamic systematic history and distinct for constructing webs with a characteristic spiral-free sector. As part of the European Reference Genome Atlas (ERGA), we generated a chromosome-level assembly for *L. thorelli* that is organized into 13 contiguous chromosomal pseudomolecules. This chromosome-level assembly encompasses 2.20 Gb and is composed of 939 contigs and 130 scaffolds, with contig and scaffold N50 values of 5.4 Mb and 167.1 Mb, respectively. This genome represents a valuable addition to the growing collection of spider genomes. With *Zygiellidae* now included among the available genomes of true orb-weavers, this is a key resource for comparative studies into the genomic basis of orb web and silk evolution.

## Introduction

*Leviellus thorelli* (Ausserer, 1871) is an orb-weaving spider species, currently classified in the family *Zygiellidae*, a small lineage recently recognised as distinct from other true orb weaving spiders ( Kuntner *et al.*, 2023). Members of this family, recognised within the subfamily *Zygiellinae*, were considered to belong to the families *Tetragnathidae* and *Araneidae*, but have since been elevated to family rank to reflect their phylogenetic position and morphological diagnosability ( Kuntner *et al.*, 2023; but see Hormiga *et al.*, 2023). The *Leviellus* genus currently includes several species distributed throughout the Palearctic region, with *L. thorelli* being one of the most widespread representatives ( World Spider Catalog, 2025). *Leviellus thorelli* occurs across Central, Southern, and South-Eastern Europe, inhabiting open woodland, grassland, and anthropogenic environments, such as gardens and buildings ( Nentwig *et al.*, 2025; World Spider Catalog, 2025). This species is a medium-sized orb-weaver that, typical of the *Zygiellidae* family, constructs vertical orb webs with a characteristic spiral-free sector; the web hub is connected to a silken-tube retreat with a signal line, reflecting a behavioural adaptation distinct from most other orb weavers ( Gregorič *et al.*, 2015). Like many spider species, *Leviellus thorelli* is a generalist predator that contributes to ecosystem balance by regulating insect populations.

From a systematic and evolutionary perspective, *L. thorelli* and its relatives are of particular interest, because they represent an early-diverging lineage within the clade of true orb weaving spiders, the *Orbipurae* ( Kuntner *et al.*, 2023). Comparative genomic data from this group can therefore provide important insights into the diversification of web architecture, web-building behaviours, silk mechanical properties, and silk gene evolution. Despite their abundance, members of the *Zygiellidae* family have previously not been represented by a reference-quality genome, limiting our understanding of genomic evolution across orb-weaving spiders. Thus, the reference genome of *L. thorelli* represents the first high-quality genome from the *Zygiellidae* family, providing a valuable resource for phylogenomic analyses aimed at refining spider systematics and taxonomy and comparative studies on the evolution of webs and silk. It will also enhance our capacity to explore genome structure and evolution both across *Orbipurae* and *Araneoidea*, providing a basis for future work in spider molecular ecology and functional genomics.

*Leviellus thorelli* is currently not classified as threatened on the IUCN Red List or any other endangered-species lists.

The generation of this reference resource was coordinated by the European Reference Genome Atlas (ERGA, https://www.erga-biodiversity.eu/) initiative’s Biodiversity Genomics Europe (BGE, https://biodiversitygenomics.eu/) project, supporting ERGA’s aim of promoting transnational cooperation to promote advances in the application of genomics technologies to protect and restore biodiversity ( Mazzoni *et al.*, 2023).

## Materials & methods

ERGA’s sequencing strategy includes Oxford Nanopore Technology (ONT) and/or Pacific Biosciences (PacBio) for long-read sequencing, along with Hi-C sequencing for chromosomal architecture, Illumina Paired-End (PE) for polishing (i.e. recommended for ONT-only assemblies), and RNA sequencing for transcriptomic profiling, to facilitate genome assembly and annotation.

### Sample and sampling information

On 07 September 2023, Matjaž Gregorič hand collected six specimens of *Leviellus thorelli* (five females, one male), at Kremenica, Slovenia (lat = 45.941472, lon = 14.548028) and Ig, Slovenia (lat = 45.964847, lon = 14.519679), which were determined based on morphology from primary taxonomic literature ( Levi, 1974). The specimens were identified by Matjaž Gregorič in Slovenia. No permit was required for the collection of the here used specimens, as communicated and confirmed by the Slovenian Ministry of Natural Resources and Spatial Planning (communicated on 16. October 2023). Specimens were hand collected, euthanized by being put alive at -80 °C, and until DNA extraction, they were preserved at -80 °C.

### Vouchering information

Physical reference materials for the here sequenced specimen have been deposited in the Slovenian Museum of Natural History, Ljubljana, Slovenia https://www.pms-lj.si/en/ under the accession number ARA8125-ARA8128.

Tissues from prosoma and from four whole organisms (proxies), as well as residual DNA and RNA, were deposited in the Leibniz Institute for the Analysis of Biodiversity Change (LIB) Biobank, Museum Koenig, Bonn, Germany, under the collection IDs ZFMK-TIS-102871, ZFMK-TIS-102868, ZFMK-TIS-102869, ZFMK-TIS-102870, ZFMK-TIS-102872, ZFMK-DNA-FD19596994, and ZFMK-RNA-FD19597045, respectively. Genitals used for future specimen identification are deposited at the Slovenian Museum of Natural History, Ljubljana, Slovenia https://www.pms-lj.si/en/.

### Genetic information

The estimated genome size, estimated by Genomes on a Tree (GoaT) ( Challis et al., 2023) by ancestral state reconstruction is 2.18 Gb. This estimated genome size corresponds to a diploid genome with a haploid number of 12 chromosomes (2n=24). All information for this species was retrieved from Genomes on a Tree ( Challis *et al.*, 2023).

### DNA/RNA processing

DNA was extracted from the prosoma of an adult female (qqLevThor3) using the Blood & Cell Culture DNA Mini Kit (Qiagen) following the manufacturer’s instructions. DNA quantification was performed using a Qubit dsDNA BR Assay Kit (Thermo Fisher Scientific), and DNA integrity was assessed using a Genomic DNA 165 Kb Kit (Agilent) on the Femto Pulse system (Agilent). The DNA was stored at +4 °C until sequenced.

RNA was extracted from prosoma using a RNeasy Mini Kit (Qiagen) according to the manufacturer’s instructions. RNA quantification was performed using the Qubit RNA BR kit, and RNA integrity was assessed using a Bioanalyzer 2100 system (Agilent) RNA 6000 Nano Kit (Agilent). The RNA was stored at -80 °C until sequenced.

### Library preparation and sequencing

For long-read whole genome sequencing, a library was prepared using the SQK-LSK114 Kit (Oxford Nanopore Technologies, ONT), which was then sequenced across two R10.4.1 flow cells on a PromethION 24 A Series instrument (ONT). A short-read whole-genome sequencing library was prepared using the KAPA Hyper Prep Kit (Roche). A Hi-C library was prepared from the prosoma of a different adult female (qqLevThor2) using the Dovetail Omni-C Kit (Cantata Bio), followed by the KAPA Hyper Prep Kit for Illumina sequencing (Roche). The RNA library was prepared using the KAPA mRNA Hyper prep kit (Roche). All short-read libraries were sequenced on a NovaSeq 6000 instrument (2x150bp, Illumina). In total 40x Oxford Nanopore, 62x Illumina WGS shotgun, and 125x HiC data were sequenced to generate the assembly.

### Genome assembly methods

The genome was assembled using the CNAG CLAWS pipeline ( Gomez-Garrido, 2024). Briefly, reads were preprocessed for quality and length using Trim Galore v0.6.7 ( http://www.bioinformatics.babraham.ac.uk/projects/trim_galore/) and Filtlong v0.2.1 ( https://github.com/rrwick/Filtlong), and initial contigs were assembled using NextDenovo v2.5.0 ( Hu *et al.*, 2024), followed by polishing of the assembled contigs using HyPo v1.0.3 ( Kundu *et al.*, 2019), removal of retained haplotigs using purge-dups v1.2.6 ( Guan *et al.*, 2020) and scaffolding with YaHS v1.2a ( Zhou *et al.*, 2024). Finally, assembled scaffolds were curated via manual inspection using Pretext v0.2.5 with the Rapid Curation Toolkit ( https://gitlab.com/wtsi-grit/rapid-curation ) to remove any false joins and incorporate any sequences not automatically scaffolded into their respective locations in the chromosomal pseudomolecules (or super-scaffolds). Summary analysis of the released assembly was performed using the ERGA-BGE Genome Report ASM Galaxy workflow ( 10.48546/workflowhub.workflow.1103.2).

## Results

### Genome assembly

**Figure 1.**
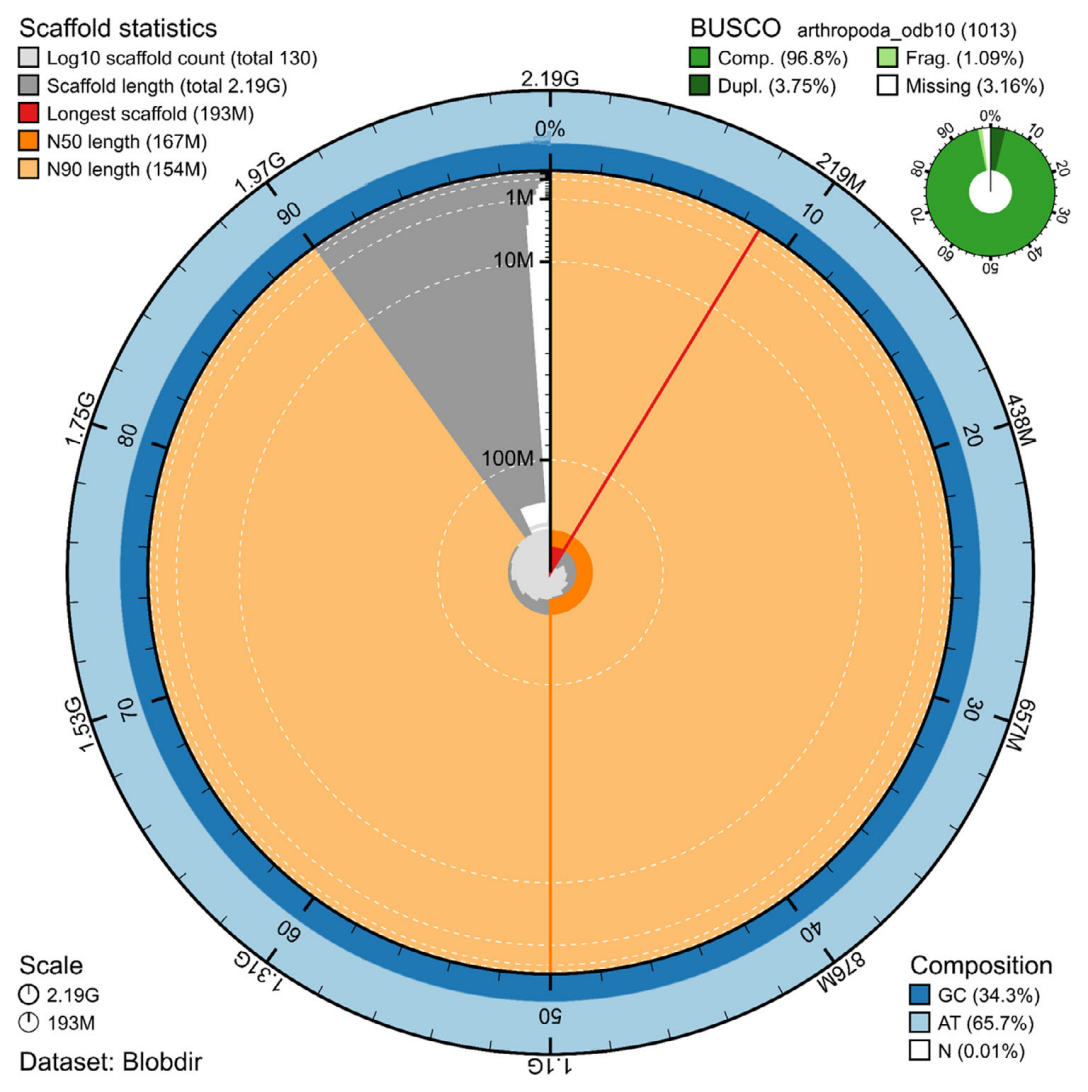
Snail plot summary of assembly statistics.

**Figure 2.**
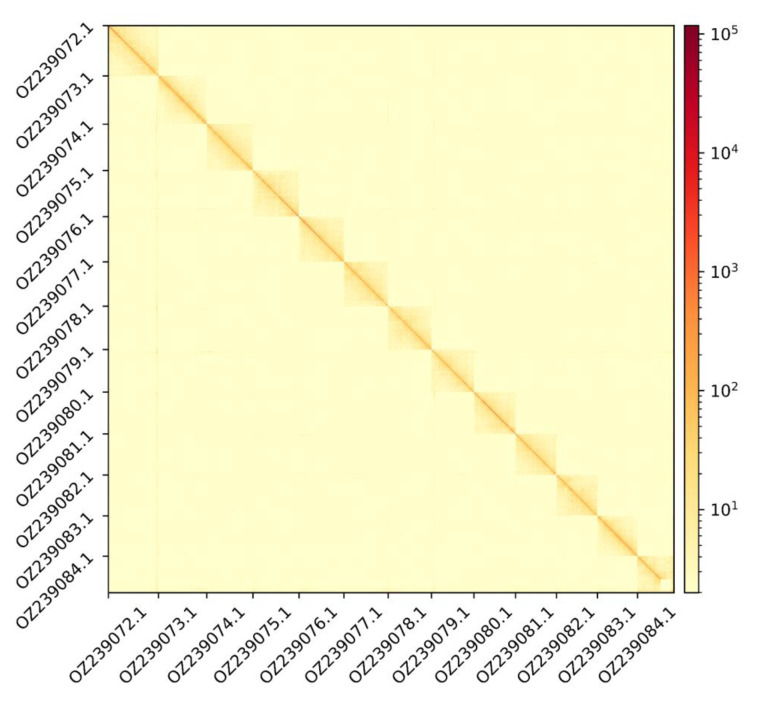
Hi-C contact map showing spatial interactions between regions of the genome.

The genome assembly has a total length of 2,190,932,837 bp in 13 superscaffolds and 17 additional unlocalized and 100 unplaced scaffolds ( Figure 1 & Figure 2), with a GC content of 34.3%. The assembly has a contig N50 of 5,371,768 bp and L50 of 119 and a scaffold N50 of 167,117,025 bp and L50 of 7. The assembly has a total of 809 gaps, totalling 161.8 kb in cumulative size. The single-copy gene content analysis using the Arthropoda database with BUSCO ( Manni *et al.*, 2021) resulted in 96.8% completeness (93.1% single and 3.8% duplicated). 93.2% of reads k-mers were present in the assembly and the assembly has a base accuracy Quality Value (QV) of 47.5 as calculated by Merqury ( Rhie *et al.*, 2020).

The main plot is divided into 1,000 size-ordered bins around the circumference, with each bin representing 0.1% of the 2,190,932,837 bp assembly. The distribution of sequence lengths is shown in dark grey, with the plot radius scaled to the longest sequence present in the assembly (193 Mb, shown in red). Orange and pale-orange arcs show the scaffold N50 and N90 sequence lengths (167,117,025 and 154,116,833 bp), respectively. The pale grey spiral shows the cumulative sequence count on a log-scale, with white scale lines showing successive orders of magnitude. The blue and pale-blue area around the outside of the plot shows the distribution of GC, AT, and N percentages in the same bins as the inner plot. A summary of complete, fragmented, duplicated, and missing BUSCO genes found in the assembled genome from the Arthropoda database (odb10) is shown in the top right.

The diagonal corresponds to intra-chromosomal contacts, depicting chromosome boundaries. The frequency of contacts is shown on a logarithmic heatmap scale. Hi-C matrix bins were merged into a 200 kb bin size for plotting.

## Author contributions

EB coordinated the project; MG collected the species; MG identified the species; MG sampled and preserved biological material and provided metadata; AsB, RM, RF, and NE provided support in sampling, shipping of biological material, metadata collection, and management; LA and MG extracted DNA, prepared libraries, and performed sequencing; FCF, FC, and JG-G performed genome assembly and curation under the supervision of TSA; CB generated the analysis and report. All authors contributed to the writing, review, and editing of this genome note and read and approved the final version.

## Data Availability

*Leviellus thorelli* and the related genomic study were assigned to Tree of Life ID (ToLID) ‘qqLevThor3’ and all sample, sequence, and assembly information are available under the umbrella BioProject PRJEB77917. The sample information is available at the following BioSample accessions: SAMEA115177265 and SAMEA115177266. The genome assembly is accessible from ENA under accession number GCA_965183905.1 and the annotated genome will be available through the Ensembl webpage ( https://projects.ensembl.org/erga-bge/). Sequencing data produced as part of this project are available from ENA at the following accessions: ERX12756489, ERX13167067, ERX14095502, and ERX14095503. Documentation related to the genome assembly and curation can be found in the ERGA Assembly Report (EAR) document available at https://github.com/ERGA-consortium/EARs/tree/main/Assembly_Reports/Leviellus_thorelli/qqLevThor3. Further details and data about the project are hosted on the ERGA portal at https://portal.erga-biodiversity.eu/data_portal/1662264.
