# Supplementary material for: ERGA-BGE reference genome of Leviellus thorelli, a common orb-weaving spider representing the Zygiellidae family
Source: Open Res Eur. 2026 Feb 10;5:365. Originally published 2025 Nov 27. [Version 2] doi: 10.12688/openreseurope.21671.2 (PMC12856242; doi:10.12688/openreseurope.21671.2)
Supplement: Peer review history

# Reviewer report: Reviewer response for version 1

**DOI:** 10.21956/openreseurope.23443.r67343

**Published:** 2026-01-29

**Authors:** Guilherme Gainett

**Affiliations:** 1 Harvard Medical School. Boston Children's Hospital, Boston, USA

The presented spider genome is a high-quality resource, and I have no major concerns.

 Great contribution to the growing number of spider genomes.

 A few comments and questions:

 -page 1 -> (Kuntner et al., 2023; in press) -> As far as I can tell, this is not in press anymore.

 -page 3: The estimated genome size, based on ancestral taxa -> could you please clarify ancestral taxa? It seems incorrect to refer to higher-level taxa or sister groups as ancestral if that's the intended meaning.

 -page 3: extracted from the thorax -> prosoma is the most used term in arachnid literature.

 -page 3: could you please clarify the statement about 12 chromosomes? How was this number estimated? 13 chromosomes are assembled later.

 Was there any contamination analysis done? A BlobToolKit GC-coverage plot could be shown to support the cleanness of the assembly. Could the small unassembled bits be due to contamination?

Are sufficient details of methods and materials provided to allow replication by others?

Yes

Is the rationale for creating the dataset(s) clearly described?

Yes

Are the datasets clearly presented in a useable and accessible format?

Yes

Are the protocols appropriate and is the work technically sound?

Yes

Reviewer Expertise:

Arachnology, Genomics, Evodevo, Zoology

I confirm that I have read this submission and believe that I have an appropriate level of expertise to confirm that it is of an acceptable scientific standard.

# Response

**Published:** 2026-01-30

**Authors:** Chiara Bortoluzzi

**Affiliations:** Swiss Institute of Bioinformatics, Lausanne, Vaud, Switzerland

We thank the reviewer for their comments. We have addressed all comments as follow:

1. We have updated the reference, which is indeed published and not in press.
2. We updated the 'Genetic information' section to clarify the concept of 'ancestral taxa' and the 12 chromosomes. 
3. We did not add any additional information on the contamination analyses, because are very well described in the EAR reported in the Genome Report.
4. We replaced the word 'thorax' throughout the manuscript as suggested by the reviewer. 

# Reviewer report: Reviewer response for version 1

**DOI:** 10.21956/openreseurope.23443.r65094

**Published:** 2026-01-02

**Authors:** Zhisheng Zhang

**Affiliations:** 1 Southwest University, Chongqing, China

The *Leviellus thorelli* reference genome provides the first high-quality genomic resource for *Zygiellidae. *This chromosome-level assembly encompasses 2.20 Gb and is composed of 939 contigs and 130 scaffolds, with contig and scaffold N50 values of 5.4 Mb and 167.1 Mb, respectively. And this genome represents a valuable addition to the growing collection of spider genomes.

 The rationale for creating the dataset was clearly described.  The protocols appropriate and the work were technically sound. 

 The sufficient details of methods and materials were provided to allow replication by others.  The datasets were clearly presented in a useable and accessible format.

Are sufficient details of methods and materials provided to allow replication by others?

Yes

Is the rationale for creating the dataset(s) clearly described?

Yes

Are the datasets clearly presented in a useable and accessible format?

Yes

Are the protocols appropriate and is the work technically sound?

Yes

Reviewer Expertise:

spider genome, evolution, adaptation

I confirm that I have read this submission and believe that I have an appropriate level of expertise to confirm that it is of an acceptable scientific standard.

# Response

**Published:** 2026-01-05

**Authors:** Chiara Bortoluzzi

**Affiliations:** Swiss Institute of Bioinformatics, Lausanne, Vaud, Switzerland

We thank the reviewer for approving the Genome Report of *Leviellus thorelli*.
